# Conjugate of PAMAM Dendrimer, Doxorubicin and Monoclonal Antibody—Trastuzumab: The New Approach of a Well-Known Strategy

**DOI:** 10.3390/polym10020187

**Published:** 2018-02-14

**Authors:** Monika Marcinkowska, Ewelina Sobierajska, Maciej Stanczyk, Anna Janaszewska, Arkadiusz Chworos, Barbara Klajnert-Maculewicz

**Affiliations:** 1 Department of General Biophysics, Faculty of Biology and Environmental Protection, University of Lodz, Pomorska 141/143, 90-236 Lodz, Poland; monika.marcinkowska@biol.uni.lodz.pl (M.M.); ewelina-s93@wp.pl (E.S.); ankuj@poczta.onet.pl (A.J.); 2 Department of Surgical Oncology, Cancer Center, Copernicus Memorial Hospital, 93-509 Lodz, Poland; macstanczyk@gmail.com; 3 Centre of Molecular and Macromolecular Studies, Polish Academy of Sciences, Sienkiewicza 112, 90-236 Lodz, Poland; achworos@cbmm.lodz.pl; 4 Leibniz-Institut für Polymerforschung Dresden e.V., Hohe Strasse 6, 01069 Dresden, Germany

**Keywords:** PAMAM dendrimer, trastuzumab, HER-2, doxorubicin, tumor targeting

## Abstract

The strategy utilizing trastuzumab, a humanized monoclonal antibody against human epidermal growth receptor 2 (HER-2), as a therapeutic agent in HER-2 positive breast cancer therapy seems to have advantage over traditional chemotherapy, especially when given in combination with anticancer drugs. However, the effectiveness of single antibody or antibody conjugated with chemotherapeutics is still far from ideal. Antibody–dendrimer conjugates hold the potential to improve the targeting and release of active substance at the tumor site. In the present study, we developed and synthesized PAMAM dendrimer–trastuzumab conjugates carrying doxorubicin (dox) specifically to cells overexpressing HER-2. ^1^HNMR, FTIR and RP-HPLC were used to characterize the products and analyze their purity. Toxicity of PAMAM–trastuzumab and PAMAM–dox–trastuzumab conjugates compared with free trastuzumab and doxorubicin towards HER-2 positive (SKBR-3) and negative (MCF-7) human breast cancer cell lines was determined using MTT assay. Furthermore, the cellular uptake and cellular localization were studied by flow cytometry and confocal microscopy, respectively. A cytotoxicity profile of above mentioned compounds indicated that conjugate PAMAM–dox–trastuzumab was more effective when compared to free drug or the conjugate PAMAM–trastuzumab. Moreover, these results reveal that trastuzumab can be used as a targeting agent in PAMAM–dox–trastuzumab conjugate. Therefore PAMAM–dox–trastuzumab conjugate might be an interesting proposition which could lead to improvements in the effectiveness of drug delivery systems for tumors that overexpress HER-2.

## 1. Introduction

According to World Health Organization (WHO) reports, breast cancer is the most common tumor among every major ethnic group of women, with almost 1.7 million new cases diagnosed in 2012 [1]. Chemotherapy is the most frequent strategy used in the battle against malignant tumors, however, it is not trouble-free. There are many reasons leading to failure of this treatment, although the inability of anticancer agents to selectively target tumor cells remains the most considerable impediment to successful chemotherapy. Thus, tumor-targeted drug delivery is one of the most thoroughly investigated research areas in terms of cancer and it is believed to be a solution to the chemotherapy limitations, including low drug efficacy and high systemic side effects [2]. 

Monoclonal antibody-based cancer treatment has been developed as one of the successful therapeutic and selective strategies [3]. The prime example of a monoclonal antibody used in this approach is humanized anti-HER2 mAb trastuzumab (Herceptin) that plays a major role in breast cancer treatment because human epidermal growth factor receptor 2 (HER-2/neu/ErbB) gene amplification or protein overexpression occurs in 20% to 25% of breast tumors. It makes it a potential candidate for targeted antibody therapy since trastuzumab blocks the HER-2 by binding to domain IV of an out-of-cell part of this protein, whereby inhibiting the excessive proliferation of tumor cells. The antibody is frequently administered together with cytotoxic agents (i.e., taxanes or anthracyclines) to increase the therapeutic efficacy [4,5]. However, the systemic toxicity of the anticancer drugs and/or decrease in the receptor recognition are still a serious problem [6]. 

New solutions are sought in terms of selective delivery of therapeutic agents. Plenty of nanoparticles such as hyperbranched polymers, liposomes, micelles or dendrimers are believed to be good candidates to play this role [7]. Among them, PAMAM dendrimers attract considerable interest. Numerous attractive features of PAMAM dendrimers such as monodispersity, nano-size and globular shape, high solubility and reactivity, biocompatibility, availability of multiple functional groups at the periphery, easy chemical modification and cavities in the interior distinguish them as unique carriers. Thus, they are an extensively studied group of nanoparticles [8,9,10,11,12,13]. Formation of stable covalent links between the surface groups of the dendrimer and a drug allows to protect the active substance in the circulatory system and transport it, through the enhanced permeability and retention effect (EPR), to the tumor environment, where, thanks to low pH, hydrolysis of covalent bonds occurs and the drug can be released. It gives a chance to reduce a dose while maintaining the same therapeutic effect. However, we decided to use PAMAM dendrimer not only as a carrier of an anticancer drug but also as a connecting link between the monoclonal antibody trastuzumab and antitumor agents. The presence of trastuzumab on the surface of the dendrimer–drug conjugate might enhance transport of the active substance directly to cells overexpressing HER-2 [14,15,16].

To verify whether the increase in conjugate selectivity for the specific type of cancer can be achieved, we synthesized a conjugate, in which we combined a protective effect of PAMAM G4 dendrimer, cytotoxic properties of doxorubicin [17] and targeted activity of trastuzumab. Our research model consisted of HER-2 positive (SKBR-3) and negative (MCF-7) human breast cancer cell lines. We performed a toxicity evaluation of PAMAM–trastuzumab and PAMAM–dox–trastuzumab conjugates compared with free trastuzumab and doxorubicin. Subsequently, we checked the rate of uptake into the cells and cellular localization of the studied compounds. Our results obtained for the conjugate PAMAM–dox–trastuzumab showed an increase in the toxic efficiency towards HER-2 positive human breast cancer cells compared to the free drug or the conjugate PAMAM–trastuzumab. To our knowledge, the proposed approach is novel and we hope that it can lead to improvements in the effectiveness of the therapy of the most common women’s cancer.

## 2. Materials and Methods

### 2.1. Materials

All chemical reagents were purchased from commercial suppliers. Solvents for the synthesis were purchased from Sigma-Aldrich (Poznan, Poland). All cell culture reagents were purchased from Gibco^®^ (Life Technologies Polska Sp. z o. o., Warsaw, Poland). Flasks and multiwell plates for in vitro studies were obtained from Nunc (Life Technologies Polska Sp. z o. o., Warsaw, Poland). Amine terminated PAMAM G4 dendrimer, doxorubicin hydrochloride, PBS (phosphate buffered saline), FBS (fetal bovine serum) and MTT (3-[4,5-dimethylthiazol-2-yl]-2,5-diphenyltetrazolium bromide) were purchased from Sigma-Aldrich. Trypan blue was purchased from Molecular Probes (Thermo Scientific™, Warsaw, Poland). Herceptin (trastuzumab) was a gift from Roche Poland. Human breast adenocarcinoma’s cell lines: HER-2 negative (MCF-7 ATCC no. HTB-22) and HER-2 positive (SKBR-3 ATCC no. HTB-30) were purchased from ATCC (LGC Standards Sp. z o. o., Lomianki, Poland).

### 2.2. Synthesis of PAMAM Doxorubicin Conjugate

Shortly, dox was dissolved in 3 mL of 0.1 M PBS at 25 °C. *cis*-Aconitic anhydride (CAA) was dissolved in 500 µL of *p*-dioxane and slowly added to the dox solution while maintaining the reaction mixture pH at 8.5. The solution was incubated with stirring for 20 min, and then for 20 min at 25 °C, in dark. Then, the reaction mixture was cooled on ice, supplemented with 100 mM HCl until pH reached 3.0 and extracted by ethyl acetate. To the resulting dox–CAA in PBS (pH 6) was added 5-fold molar excess of *N*-(3-Dimethylaminopropyl)-*N′*-ethylcarbodiimide hydrochloride (EDC), and the mixture was stirred at 20 °C for 0.5 h, in dark. PAMAM G4 was dissolved in 1 mL of PBS, pH 6.0, dox–CAA was added. The mixture was incubated with intensive stirring at 25 °C, pH 7.8, for 12 h. The PAMAM G4–dox was purified by ultrafiltration on an Amicon Ultra-3K (molecular weight cut-off, MWCO = 3 kDa, Sigma-Aldrich, Poznan, Poland). ^1^HNMR and FTIR was used to analyze the purity of products and to ascertain the level of PAMAM and doxorubicin conjugation. ^1^HNMR spectra were recorded on Bruker Avance III DRX-600 and 500 MHz spectrometers (Poznan, Poland), using deuterated D_2_O as solvents. The FTIR spectra were collected with a FTIR ATI Mattson Spectrometer Spectrum (Middleton, MA, USA) and samples were measured as thin film in KBr crystals. The analytical data can be found in the Supplementary Materials.

### 2.3. Synthesis of PAMAM–dox–trastuzumab Conjugate

The synthesis of PAMAM doxorubicin trastuzumab conjugate was performed using a method invented by us (patent pending P.421439)*.*

*Activation of trastuzumab:* SMCC was dissolved in a small volume of DMF, and diluted by adding 0.1 M PBS (phosphate buffered saline) pH 7.6, which contains 5 mM EDTA to obtain 1 mg/mL. The solution was added to trastuzumab. The mixture was incubated for 1 h at room temperature (RT). In the next steps crude mixture was purified and buffer-exchanged into PBS pH 7.0, with Amicon Ultra-30 K column (MWCO = 30 kDa).

*Introduction of thiol groups for the PAMAM G4 dendrimer surface:* Traut’s reagent converts primary amine into thiol in the range of pH 7–10, however its half-life in solution decreases as the pH increases. Modification with Traut’s reagent (2-iminothiolane) is very efficient and occurs rapidly at slightly basic pH. To introduce thiol groups into G4 dendrimer surface, the primary amine groups were reacted with a 10:1 mole excess of Traut’s reagent in 0.1 M PBS buffer, at room temperature under N_2_ for 1 h pH 8.0. Thiolated PAMAM G4 was purified and buffer exchanged into PBS, pH 7.0 by ultrafiltration on an Amicon Ultra-3 K column. 

*The reaction of the modified PAMAM G4 dendrimer with the activated trastuzumab:* Derivatized trastuzumab was reacted with thiolated PAMAM G4 dendrimer at a 1:12 molar ratio. The reaction was conducted in PBS, pH 7.0 at 25 °C for 24 h. Finally, PAMAM–trastuzumab conjugate was purified from excess thiolated PAMAM G4 by Amicon Ultra-30 K (MWCO 30 kDa). The final stoichiometric ratio for PAMAM–dox–trastuzumab conjugate was 1:1:1.

Reverse phase high performance liquid chromatography (RP-HPLC) was used to analyze the purity of products and to ascertain the level of PAMAM and trastuzumab conjugation. Solvents used for HPLC analysis were at the HPLC grade; iPrOH, MeOH, MeCN was from Sigma-Aldrich (Poznan, Poland), trifluoroacetic acid from J.T.Baker® (9470) and Milli-Q water. All experiments were performed on two FPLC/HPLC systems: (1) AKTA Purifier two pumps system equipped with UV-900 monitoring (GE Healthcare Life Sciences, Pittsburgh, PA, USA), pH and conductivity probe and fraction collector Frac-920 (GE Healthcare Life Sciences, Pittsburgh, PA, USA). Analysis using AKTA was performed at room temperature 25 °C. (2) Shimadzu Prominence UFLC system equipped with LC-20AD isocratic pumps (Shimadzu Scientific Instruments Incorporated, Columbia, MD, USA) with RF-20A fluorescence detector (Shimadzu Scientific Instruments Incorporated, Columbia, MD, USA), SPD-M20A diode array detector (Shimadzu Scientific Instruments Incorporated, Columbia, MD, USA) for UV–Vis monitoring and CTO-20ASvp column oven (Shimadzu Scientific Instruments Incorporated, Columbia, MD, USA) that was setup at 75 °C. Initially, SOURCE uRPC C2/C18 ST 4.6/100 column (Pharmacia Biotech, SanJose, CA, USA) was used, but it appeared to be too hydrophobic for dendrimer and antibody analysis, therefore for all presented results Jupiter 4u Proteo 90A 2.0/100 column (Phenomenex Inc., Torrance, CA, USA) was used. The analytical data can be found in the Supplementary Materials.

### 2.4. Cell Culture

HER-2 negative human breast adenocarcinoma (MCF7) cell line was grown in DMEM medium supplemented with GlutaMAX and 10% (*v*/*v*) fetal bovine serum (FBS). HER-2 positive human breast adenocarcinoma (SKBR3) cell line was grown in McCoy’s 5 medium supplemented GlutaMAX and 10% (*v*/*v*) fetal bovine serum (FBS). Cells were cultured in T-75 culture flasks in a humidified atmosphere containing 5.0% CO_2_ at 37 °C and subcultured every 2 or 3 days. Cells were harvested and used in experiments after obtaining 80–90% confluence. The number of viable cells was determined by the trypan blue exclusion assay with the use of Countess Automated Cell Counter (Invitrogen). Cells were seeded in flat bottom 96-well plates at a density of 2.0 × 10^4^ cells/well in 100 μL of an appropriate medium. After seeding, plates were incubated for 24 h in a humidified atmosphere containing 5.0% CO_2_ at 37 °C to allow cells to attach to the plates.

### 2.5. Determination of Cytotoxicity 

The influence of the PAMAM dendrimer conjugates and pure doxorubicin on the cell viability was determined with the use of the MTT-assay. Briefly, to the 96-well plates containing cells at the density of 2.0 × 10^4^ cells/well in medium different concentrations of all compounds were added. Cells were incubated with the dendrimer for 24 h in a 37 °C humidified atmosphere containing 5.0% CO_2_. After the incubation period, cells were washed with phosphate buffered saline (PBS). Next, 50 µL of a 0.5 mg/mL solution of MTT in PBS was added to each well and cells were further incubated under normal culture conditions for 4 h. After incubation, the residue MTT solution was removed and the obtained formazan precipitate was dissolved in DMSO (100 µL/well). The conversion of the tetrazolium salt (MTT) to a colored formazan by mitochondrial and cytosolic dehydrogenases is a marker of cell viability. Before the absorbance measurement, plates were shaken for 1 min and the absorbance at 570 nm was measured on the PowerWave HT Microplate Spectrophotometer (BioTek, Winooski, VT, USA).

### 2.6. Cellular Uptake Detection

In vitro uptake studies were carried out using fluorescent doxorubicin and PAMAM–dox–trastuzumab conjugate. The compounds were added at a concentration of 1 µM to the 12-well plates containing cells at the density of 1.5 × 10^4^ cells/well (SKBR-3, MCF-7). Cells were incubated with the compounds for a specific time in a range from 1 h to 48 h in humidified atmosphere containing 5.0% CO_2_ at 37 °C. After the appropriate incubation period, cells were washed with PBS, suspended in 500 µL of medium and immediately analyzed with a Becton Dickinson LSR II flow cytometer (BD Biosciences, San Jose, CA, USA) using a blue laser (488 nm) and PE bandpass filter (575/26 nm).

### 2.7. Confocal Microscopy

Confocal microscopy images were obtained under 6300× magnification with Zeiss LSM 780 microscope equipped with 405 nm laser diode and InTune excitation laser system (Carl Zeiss Inc., Oberkochen, Germany). Cells at the density of 1 × 10^4^ cells/well (SKBR-3) and 0.75 × 10^4^ cells/well (MCF-7) were seeded on 96-well glass-bottom plates and incubated with 1 µM doxorubicin or PAMAM–dox–trastuzumab conjugate for 24 h in 37 °C humidified atmosphere containing 5.0% CO_2_. After the incubation, cells were cooled on ice and washed once with cold phosphate buffered saline (PBS) to inhibit endocytosis. Cell nuclei were stained with DAPI in PBS for 10 min. Stained cells were imaged to visualize fluorescence of doxorubicin in far-red channel (excitation 595 nm, emission 600–740 nm) and nuclei in blue channel (excitation 405 nm, emission 410–470 nm).

### 2.8. Statistical Analysis

Data were expressed as mean ± SD. Analysis of variance (ANOVA) with the Tukey post hoc test was used for results comparison. All statistics were calculated using the Statistica software (version12, StatSoft, Tulsa, OK, USA, 2013), and *p* values < 0.05 were considered significant.

## 3. Results and Discussion

### 3.1. Synthesis and Characterization of PAMAM–trastuzumab and PAMAM–dox–trastuzumab Conjugates

**Figure 1:**
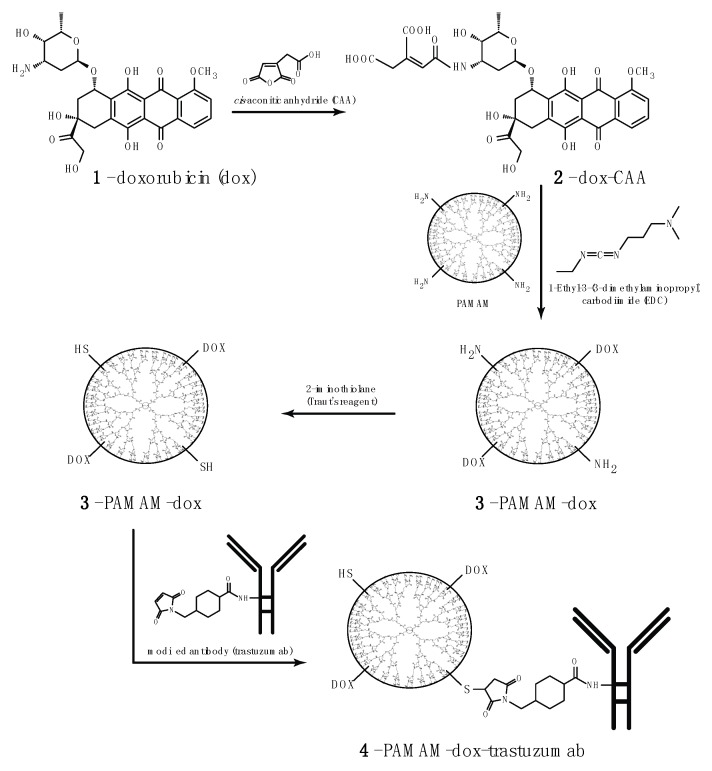
Synthesis of PAMAM–dox–trastuzumab conjugate.

The main drawback of most breast cancer treatments is high systemic toxicity, which leads to side effects. To overcome this nonspecific cytotoxicity, we synthesized a conjugate consisting of three components, each of them playing a different role: (1) trastuzumab provides specificity against human epidermal growth factor receptor 2 (HER-2) that overexpresses in various cancer, including breast cancer; (2) doxorubicin provides cytotoxic effect; and (3) PAMAM dendrimer protects the whole conjugate in the circulatory system and, when linked with doxorubicin via pH-sensitive linker, provides drug release in tumor environment. Yabbarov et al. confirmed the dependence of release of doxorubicin on decreasing pH [18]. It has been observed that pH-dependent linkage is hydrolyzed and the drug is released in the environment of the tumor, which allows for the controlled administration of the active substance in the chosen site using the natural properties of tumor cells: fast metabolism and the acidic pH. We decided to combine doxorubicin and PAMAM dendrimer using *cis*-aconitic anhydride (CAA) [19]. Figure 1 presents the steps of PAMAM–dox–trastuzumab conjugate synthesis.

The chemical structure of PAMAM–dox was characterized by ^1^HNMR analysis and FTIR spectroscopy. The analytical data can be found in the Supplementary Materials.

In the next step, the PAMAM–dox conjugate was connected to the monoclonal antibody. To accomplish this, we used a succinimidyl 4-(*N*-maleimidomethyl) cyclohexane-1-carboxylate (SMCC) linker. The SMCC is a convenient crosslinking agent for the amino and thiol groups. The NHS esters react with primary amines to form stable amide bonds and maleimide part reacts with sulfhydryl groups to form stable thioethers. Therefore, to carry out this reaction, we had to modify amine PAMAM dendrimer groups into thiols. For this purpose, the Traut’s reagent was used. Modification with Traut’s reagent (2-iminothiolane) is very efficient and rapidly. It occurs at slightly basic pH [20].

**Figure 2:**
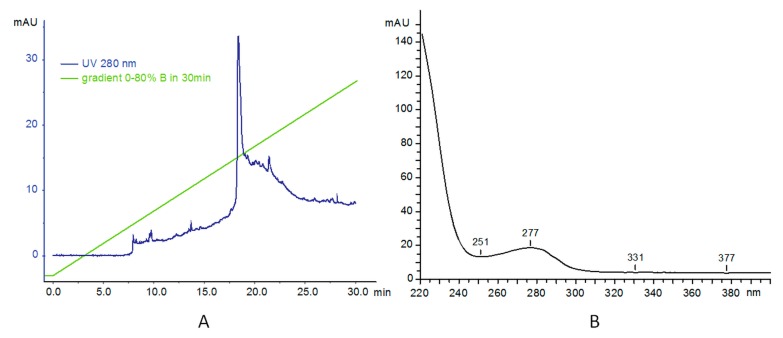
(**A**) RP-HPLC profile of trastuzumab; and (**B**) UV profile of the main signal at 18 min.

To characterize the conjugates, we used FPLC/HPLC analysis. Reverse phase high performance liquid chromatography (RP-HPLC) was used to analyze the purity of products and to ascertain the level of PAMAM and trastuzumab conjugation. The water/acetonitrile elution system was used initially, but better performance was achieved with following: A: 0.1% TFA in water; and B: 70% iPrOH, 20% MeCN, and 0.1% TFA in water. The typical gradient contained 0–80% B for 30 min 80–100% B in 5 min, 100% B for 10 min and 100–0% B in 5 min, as shown in Figure 2. Samples were typically injected as 20–100 µg of material suspended in 100 µL buffer A. As reported before, main absorption for PAMAM dendrimer is at 214 nm and this wavelength was used as well as 280 nm for the protein. However, we also monitored at 254 nm for a potential contamination. Additionally, Shimadzu diode array system provided us with UV profiles (200–600 nm was used). This allowed us to ascertain the purity of PAMAM dendrimer, absorbing at 220 nm as also reported before. The analytical data can be found in the Supplementary Materials.

In the second step, analysis of trastuzumab was carried out. Analysis was performed on UFLC system (two LC-20ADXP isocratic pumps, a CTO-20AS column oven with DIOD array UV–Vis monitoring) at 75 °C. The elution system was as before: A: 0.1% TFA in water; B: 70% iPrOH, 20% MeCN, and 0.1% TFA in water. In the gradient 0–80% for 30 min, the main product (as monitored at 280 nm) appeared at 18.3 min. The UV profile of the main signal shows absorbance at 277 nm, as expected characteristic for proteins (Figure 2B).

**Figure 3:**
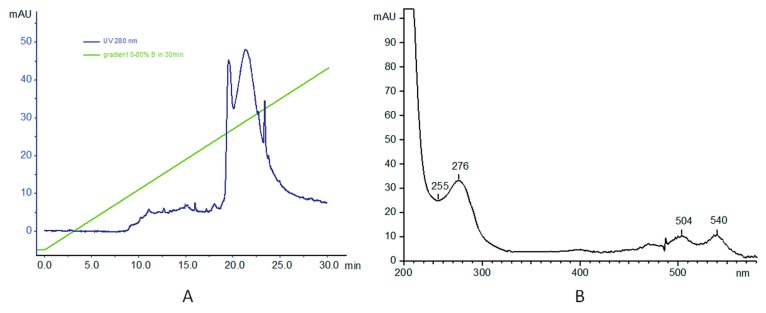
(**A**) RP-HPLC profile of PAMAM–dox–trastuzumab conjugate analysis; and (**B**) UV profile of the signal at 21 min.

Finally, PAMAM–dox–trastuzumab conjugate was analyzed. Profile analysis showed an absorption at 280 nm. Analysis was carried out as before at 75 °C, in: A: 0.1% TFA in water; and B: 70% iPrOH, 20% MeCN, and 0.1% TFA in water; and the gradient 0–80% for 30 min, injected 100 µg. The UV profile of the signal at 21 min shows three signals (Figure 3B). As expected, the absorbance signals at 276, 504 and 540 nm are characteristic for protein (the first) and doxorubicin (the last two signals).

### 3.2. In Vitro Studies

**Figure 4:**
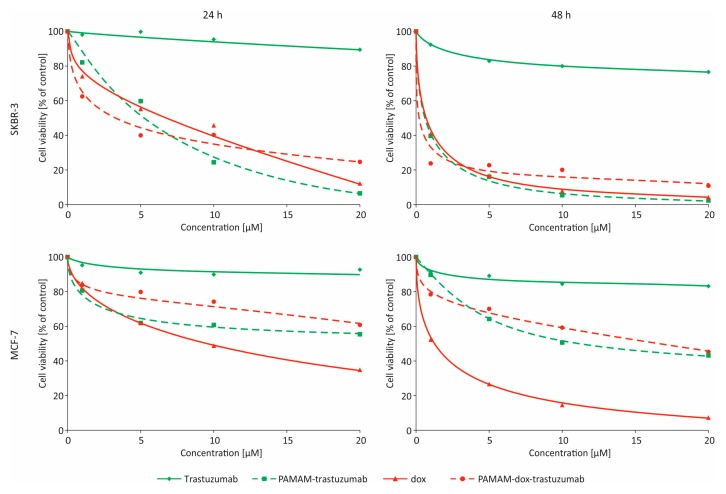
Influence of trastuzumab (green rhombus), doxorubicin (red triangles), PAMAM–trastuzumab (green squares) and PAMAM–dox–trastuzumab (red spheres) conjugates on the viability of MCF-7 and SKBR-3 cells assessed by MTT assay.

Two breast cancer cell lines (MCF-7 and SKBR-3) were used to study the anticancer activity of PAMAM–trastuzumab and PAMAM–dox–trastuzumab conjugates compared with free trastuzumab and doxorubicin. The cell lines were selected for their immunological profile. SKBR-3 cells are HER-2 positive while MCF-7 cells are HER-2 negative. Cytotoxicity was assessed using MTT assay to understand contribution of the above-mentioned compounds in targeting HER-2 positive breast cancer (Figure 4). The measurements were made after 24 and 48 h of incubation and additionally after 24 h incubation with the drug and 24 h incubation after removal of the drug (24–24 h). The addition of this incubation variant allows the assessment of cell damage and mortality after the drug is removed from the system.

The cytotoxicity profile clearly indicates that free trastuzumab was inefficient in the tested concentration range but the conjugation of antibody with PAMAM dendrimer improved that cytotoxic effect. The observed effect was more pronounced for SKBR-3 cells than MCF-7 cells due to selective adhesion of the conjugate to cells overexpressing the HER-2. Moreover, the addition of doxorubicin, which is often administered in alternating cycles with trastuzumab, to PAMAM–trastuzumab conjugate enhanced the therapeutic effect and the selectivity, which was particularly noticed after 48 h of incubation. The IC_50_ values (Table 1) for SKBR-3 cells demonstrated that PAMAM–dox–trastuzumab conjugate (0.003 ± 0.002 µmol/L) showed rise in selectivity and therapeutic effect when compared not only to PAMAM–trastuzumab conjugate (0.41 ± 0.06 µmol/L) but also to the free drug (0.342 ± 0.127 µmol/L). It indicates that there is a possibility of dose reduction while maintaining a therapeutic effect and selectivity, which can protect from cardiotoxicity caused by doxorubicin. Our results obtained for the conjugate PAMAM–dox–trastuzumab are unique because they show a synergistic effect and an increase in the toxic efficiency towards to HER-2 positive cells compared to the free drug or the conjugate PAMAM–trastuzumab and a decrease in the toxic efficiency towards to HER-2 negative cells. Recently, the effectiveness of dendrimer with conjugated monoclonal antibody and anticancer drug (docetaxel) has been also observed by Kulhari et al. [21]. These results demonstrate that trastuzumab can specifically target and deliver anticancer to HER2-positive cells.

**Figure 5:**
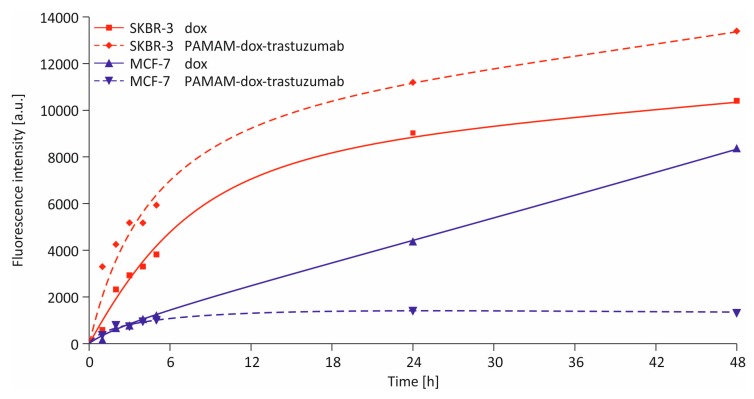
Cellular uptake of free doxorubicin and PAMAM–dox–trastuzumab conjugate at a concentration of 1 µM by MCF-7 (blue triangles) and SKBR-3 (red rhombus/squares) cells after incubation for 1, 2, 3, 4, 5, 24, and 48 h.

To analyze cellular uptake of doxorubicin and PAMAM–dox–trastuzumab conjugate by flow cytometry, cells were incubated with the compounds at concentration of 1 µM. Incubation times varied from 1 h to 48 h. The fluorescence could be observed because doxorubicin itself has an intrinsic fluorescence, which paradoxically makes it an imaging agent. All tested cell lines accumulated both compounds rapidly, although the largest amount was observed in SKBR-3 cells (Figure 5). In the case of MCF-7 cells, after 48 h incubation, the fluorescence intensity, which is directly proportional to the doxorubicin concentration, was considerably lower after treatment with PAMAM–dox–trastuzumab conjugate than with free doxorubicin. As expected, the effect was quite the opposite in terms of SKBR-3 cells, where uptake of PAMAM–dox–trastuzumab conjugate was higher than the free drug, which is in great agreement with our results obtained by MTT assay. This outcome corresponds to previous reports that PAMAM–methotrexate–trastuzumab specifically internalize in breast cancer cell line overexpressing HER-2 (MCA207-HER-2) [20]. This finding suggests that synthesis of HER-2-targeted dendrimer–drug conjugate can find applications in tumor-targeted drug delivery.

**Figure 6:**
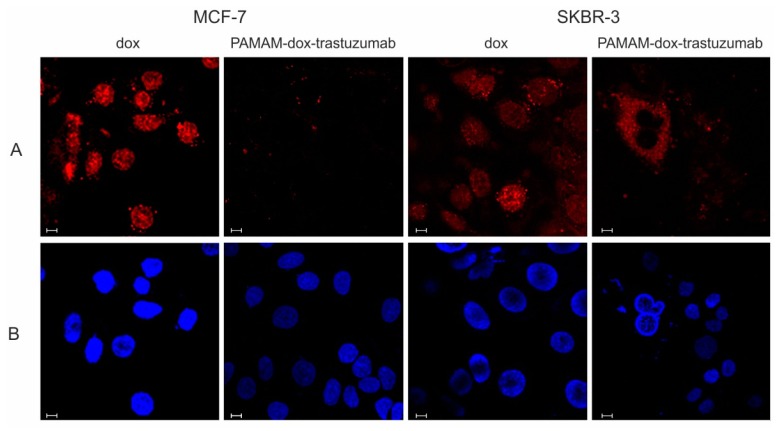
Confocal images of MCF-7 and SKBR-3 cells treated with 1 µM free doxorubicin and PAMAM–dox–trastuzumab conjugate for 24 h. Following doxorubicin and conjugate accumulation (red channel) (**A**), cells were rinsed once with PBS and stained with DAPI to visualize cell nucleus (blue channel) (**B**). The size of the scale bar is 10 nm.

The increase in fluorescence intensity observed upon incubation of cells with the drug and conjugate may occur due to two processes: an uptake of the compounds within the cells or binding of them to the outer layer of cell membranes. To make certain that analyzed compounds actually localized in the cells, confocal microscopy was used as a visualization technique. Again, this method was based on the fluorescence of the doxorubicin. The concentration of the doxorubicin remained the same and equaled 1 µM. Confocal images are presented in Figure 6.

Images confirm internal localization of the compounds. As expected, free doxorubicin was localized internally in all tested cell lines but when it comes to PAMAM–dox–trastuzumab conjugate, some differences in localization can be observed between tested cell lines. In the case of SKBR-3 cells, conjugate was internalized to cell nucleus while in MCF-7 cells was almost not present there, which was in parallel with our cytotoxicity and uptake studies. Furthermore, trastuzumab labeled by doxorubicin was visible on the surface of the cells and in the cytoplasm. Interestingly, the interaction of the monoclonal antibody and only a small amount of doxorubicin, which binds to nucleic acids, was needed to ensure high efficacy of the conjugate. To the best of our knowledge, this the first example of the study of the localization of PAMAM–dox–trastuzumab in the cells. Others researchers were investigating the internalization of dendrimer–antibody conjugate in the cells. Miyano et al. used PAMAM G6 dendrimers modified on the surface with lysine and glutamic acid (KG6E) and with attached trastuzumab [22]. Studies with this conjugate were also carried out on two human breast cancer cell lines: SKBR-3 (HER-2 positive) and MCF-7 (HER-2 negative). After 1 h incubation at 4 °C, KG6E–trastuzumab–Alexa Fluor 488 conjugate was selectively bound to SKBR-3 cells, rather than MCF-7. Moreover, the conjugate was internalized to lysosomes. In other studies, trastuzumab was covalently linked to a PAMAM dendrimer via bifunctional polyethylene glycol (PEG) and tested towards to two cell lines BT474 (HER-2 positive) cells and MCF-7 (HER-2 negative) cells [23]. These in vitro studies demonstrated that PAMAM–trastuzumab conjugate was taken up by HER-2-overexpressing BT474 cells more efficiently than MCF-7 cells that expressed lower levels of HER-2. Co-localization experiments indicated that trastuzumab-conjugated PAMAM was located in the cytoplasm. These findings provide utilizable information for design conjugate for targeted therapy. 

## 4. Conclusions

In summary, we have successfully synthesized and characterized HER-2 targeted PAMAM–dox–trastuzumab conjugate. The in vitro cytotoxicity, cellular uptake and internalization studies indicate that this conjugate is a promising carrier for HER-2-expressing tumor-selective delivery. The selectivity is not only due to trastuzumab that binds to human epidermal growth factor receptor 2 but also due to pH-sensitive linker between dendrimer and doxorubicin that in the tumor environment breaks allowing to drug release. PAMAM–dox–trastuzumab conjugate can find application in the drug delivery system and enhance the therapeutic index of anticancer drug while reducing its dose.

## Figures and Tables

**Table 1:** Comparison of IC_50_ value for trastuzumab, doxorubicin, PAMAM–trastuzumab and PAMAM–dox–trastuzumab conjugates in two breast cancer cell lines. The IC_50_ values are presented as mean ± S.D. of three experiments.

	MCF-7 24 h	MCF-7 24 h–24 h	MCF-7 48 h	SKBR-3 24 h	SKBR-3 24 h–24 h	SKBR-3 48 h
Trastuzumab	>100	>100	>100	>100	>100	>100
PAMAM–trastuzumab	32.46 ± 4.47 *	11.31 ± 4.06 *	11.92 ± 4.08 *	4.29 ± 0.06 *	2.81 ± 1.52 *	0.41 ± 0.06 *
Dox	9.20 ± 1.23	1.37 ± 1.61	1.10 ± 1.23	0.77 ± 0.16	0.19 ± 0.08	0.34 ± 0.13
PAMAM–dox–trastuzumab	38.40 ± 5.73	8.17 ± 4.85	14.86 ± 5.37	2.81 ± 0.74	0.14 ± 0.04	0.003 ± 0.002 *

* Statistically significant difference towards free drug at *p* < 0.05.

## Supplementary Materials

The following are available online at http://www.mdpi.com/2073-4360/10/2/187/s1, Figure S1: The 1HNMR spectrum of PAMAM-dox conjugate, Figure S2: The FTIR spectrum of PAMAM-dox, Figure S3: RP-HPLC profile of PAMAM G4, trastuzumab and PAMAM–dox–trastuzumab conjugate analysis.
