# Supplementary material for: Conjugate of PAMAM Dendrimer, Doxorubicin and Monoclonal Antibody—Trastuzumab: The New Approach of a Well-Known Strategy
Source: Polymers (Basel). 2018 Feb 14;10(2):187. doi: 10.3390/polym10020187 (PMC6414888; doi:10.3390/polym10020187)
Supplement: Supplementary file 1 [file polymers-10-00187-s001.doc]

**Conjugate of PAMAM dendrimer, doxorubicin and monoclonal antibody – trastuzumab: The new approach of a well–known strategy**

Monika Marcinkowska^1^, Ewelina Sobierajska^1^, Maciej Stanczyk^2^, Anna Janaszewska^1^, Arkadiusz Chworos^3^, Barbara Klajnert-Maculewicz^1,4^

*^1^ Department of General Biophysics, Faculty of Biology and Environmental Protection, University of Lodz, Pomorska 141/143, 90-236 Lodz, Poland*

*^2^ Department of Surgical Oncology, Cancer Center, Copernicus Memorial Hospital, Lodz, Poland*

*^3^ Centre of Molecular and Macromolecular Studies, Polish Academy of Sciences, Sienkiewicza 112, 90-236 Lodz, Poland*

*^4^ Leibniz-Institut für Polymerforschung Dresden e.V., Hohe Strasse 6, 01069 Dresden, Germany*

**Abstract**

The strategy utilizing trastuzumab, a humanized monoclonal antibody against human epidermal growth receptor 2 (HER-2), as a therapeutic agent in HER-2 positive breast cancer therapy seems to have advantage over traditional chemotherapy especially when given in combination with anticancer drugs. However, the effectiveness of single antibody or antibody conjugated with chemotherapeutics is still far from ideal. Antibody-dendrimer conjugates hold the potential to improve the targeting and release of active substance at the tumor site. In the present study, we developed and synthesized PAMAM dendrimer-trastuzumab conjugates carrying doxorubicin (dox) specifically to cells overexpressing HER-2. ^1^HNMR, FTIR and RP-HPLC was used to analyze the charakterize the products and to analyze their purity. Toxicity of PAMAM-trastuzumab and PAMAM-dox-trastuzumab conjugates compared with free trastuzumab and doxorubicin, towards HER-2 positive (SKBR-3) and negative (MCF-7) human breast cancer cell lines was determined using MTT assay. Furthermore, the cellular uptake and cellular localization were studied by flow cytometry and confocal microscopy, respectively. A cytotoxicity profile of above mentioned compounds indicated that conjugate PAMAM-dox-trastuzumab was more effective when compared to free drug or the conjugate PAMAM-trastuzumab. Moreover, these results reveal that trastuzumab can be used as a targeting agent in PAMAM-dox-trastuzumab conjugate. Therefore PAMAM-dox-trastuzumab conjugate might be an interesting proposition which could lead to improvements in the effectiveness of drug delivery systems for tumors that overexpress HER-2.

*Synthesis of PAMAM doxorubicin conjugate*

^1^HNMR and FTIR was used to analyze the purity of PAMAM-dox conjugate. ^1^HNMR spectra were recorded on Bruker Avance III DRX-600 and 500 MHz spectrometers, using deuterated D_2_O as solvents. The FTIR spectra were collected with a FTIR ATI Mattson Spectrometer Spectrum and samples were measured as thin film in KBr crystals.

As shown in Figure S1, the peaks at 7.7 ppm resulted from aryl groups and 5.15 ppm, 0.94 ppm for alkyl group of the doxorubicin. The 6.5 ppm signal refers to the CAA linker. The peaks at 2.2-3.2 ppm correspond to protons of PAMAM dendrimer.


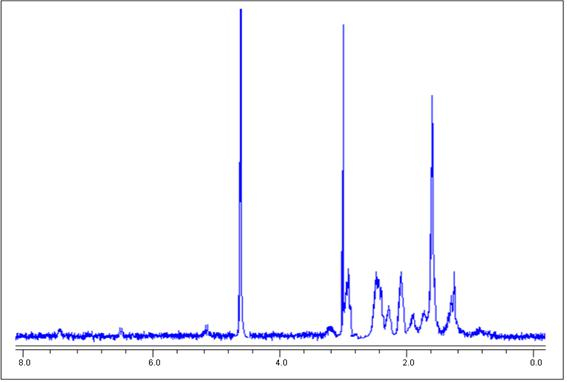


**Figure S1.** The ^1^HNMR spectrum of PAMAM-dox conjugate

According to FTIR, the typical peak attributed to NH groups of the conjugate was observed at 1637 cm^-1^. The broad band at 3416 cm^-1^ corresponds to O-H. The N-H bond from PAMAM appears at 1550, 1465, 1364 cm^-1^. The bands at 2849, 2837 cm^-1^ and 1153 cm^-1^ are attributed to C-H and C-O-C groups of doxorubicin respectively. In the IR spectrum of PEG- dox the peak at 3416 cm^-1^ is attributed to OH and N-H groups. The FTIR spectrum of PAMAM-dox is shown on the Figure S2.


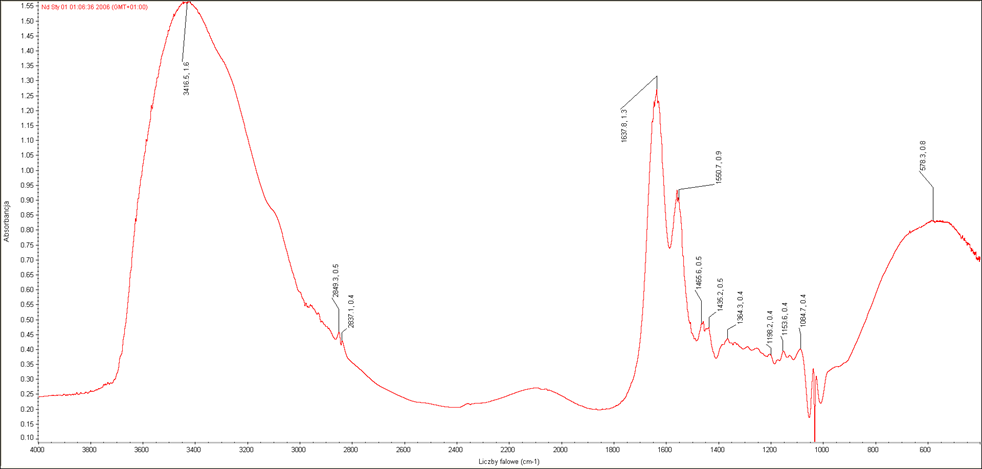


**Figure S2.** The FTIR spectrum of PAMAM-dox

*2. Synthesis of PAMAM-dox-trastuzumab conjugate*

Reverse phase high performance liquid chromatography (RP-HPLC) was used to analyze the purity of products and to ascertain the level of PAMAM, PAMAM-dox and trastuzumab conjugation (Figure S3). Solvents used for HPLC analysis were at the HPLC grade; ^i^PrOH, MeOH, MeCN was from Sigma-Aldrich, trifluoroacetic acid from J.T.Baker (9470) and Milli-Q water. All experiments were performed on two FPLC/HPLC systems: (1) AKTA Purifier two pumps system equipped with UV-900 monitoring, pH and conductivity probe and fraction collector Frac-920. Analysis using AKTA was performed at room temperature 25^o^C, (2) Shimadzu Prominence UFLC system equipped with LC-20AD isocratic pumps with RF-20A fluorescence detector, SPD-M20A diode array detector for UV-Vis monitoring and CTO-20ASvp column oven that was setup at 75^o^C. Initially SOURCE uRPC C2/C18 ST 4.6/100 column was used, but it appeared to be too hydrophobic for dendrimer and antibody analysis, therefore for all presented results Jupiter 4u Proteo 90A 2.0/100 column was used.

Figure S3. shows RP-HPLC profile of PAMAM analysis performed on AKTA Purifier system with constant UV monitoring at 3 wavelengths (220, 240 and 280 nm), pH and conductivity at 25^o^C. The elution system was optimized and finally contained A: 0.1% TFA in water, B: 70% ^i^PrOH, 20% MeCN, 0.1% TFA in water. In the gradient 0-80% for 30 min the main product (PAMAM dendrimer) appeared at 5.8 min with the purity estimated for 96.8%.


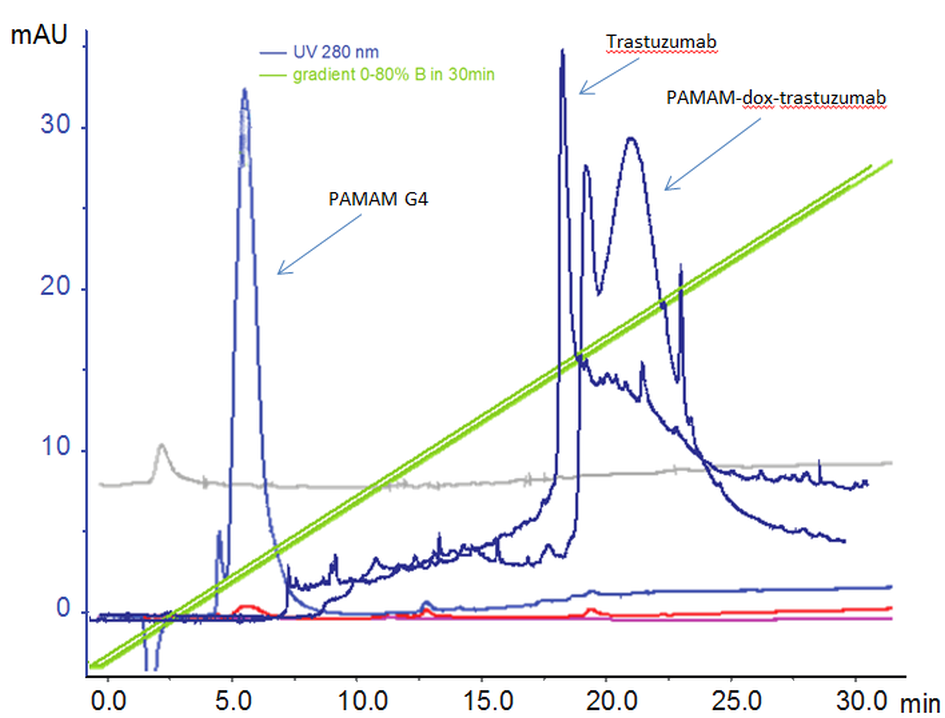


**Figure S3**. RP-HPLC profile of PAMAM G4, trastuzumab and PAMAM-dox-trastuzumab conjugate analysis
